# Supplementary material for: The interactome of intact mitochondria by cross-linking mass spectrometry provides evidence for coexisting respiratory supercomplexes
Source: Mol Cell Proteomics. 2017 Dec 8;17(2):216–32. doi: 10.1074/mcp.RA117.000470 (PMC5795388; doi:10.1074/mcp.RA117.000470)
Supplement: Supplemental Data [file supp_RA117.000470_133922_0_supp_23978_fzffwf.zip › spectra_annotation/mito_spectra_annotation/113-1-1-1-9-1.pdf]

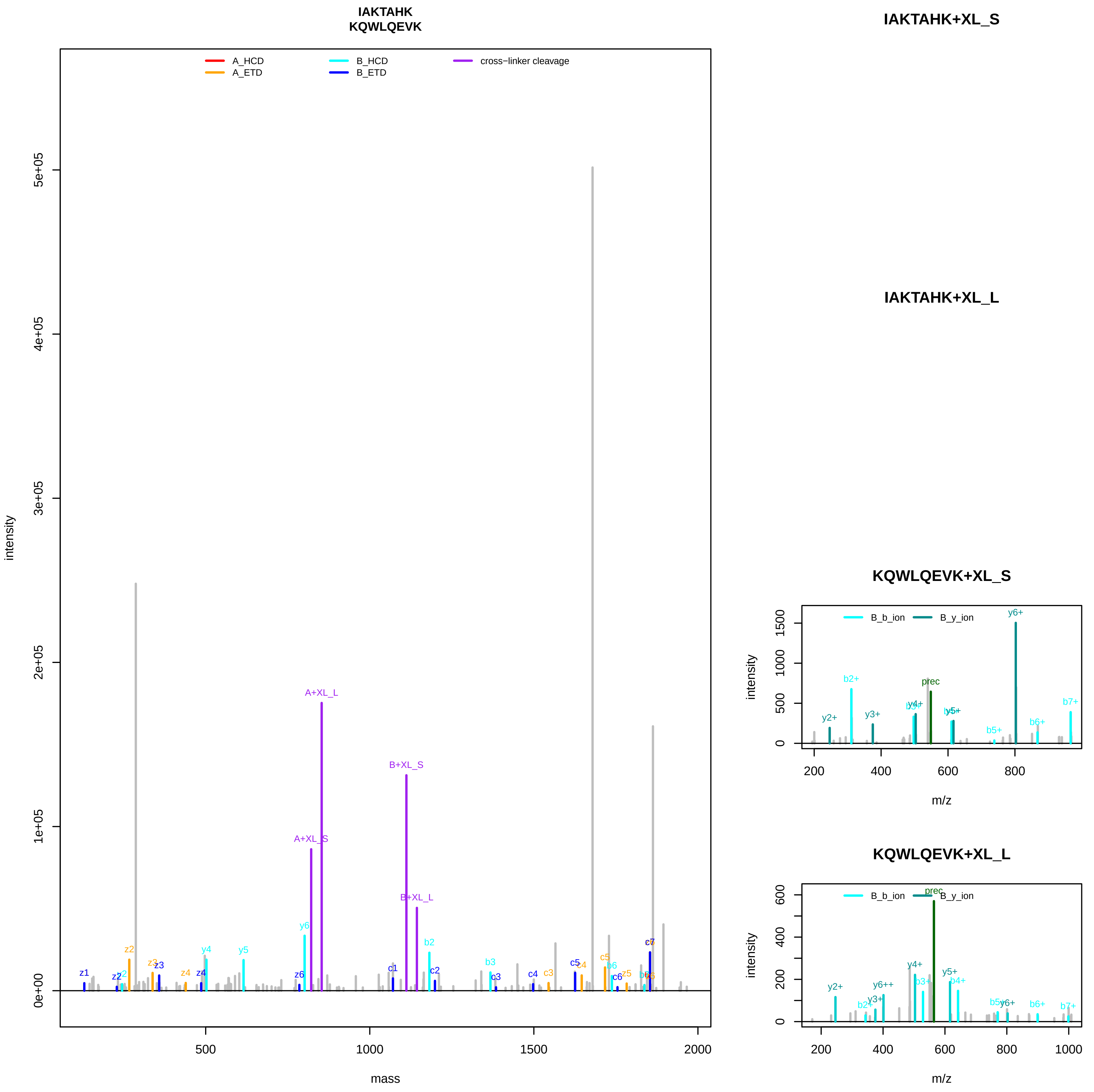
IAKTAHK
KQWLQEVK
A_HCD
A_ETD
B_HCD
B_ETD
cross−linker cleavage
intensity
mass
0e+00
1e+05
2e+05
3e+05
4e+05
5e+05
500
1000
1500
2000
A+XL_L
A+XL_S
B+XL_S
B+XL_L
z1 z2 z3 z4 z6
z2 z3 z4 z5 z6
y2 y4 y5 y6
b2 b3 b6
c1 c2 c3 c4 c5 c6 c7
c3 c4 c5 c7
IAKTAHK+XL_S
IAKTAHK+XL_L
KQWLQEVK+XL_S
B_b_ion
B_y_ion
intensity
m/z
0
500
1000
1500
200
400
600
800
y2+ b2+ y3+ b3+ y4+ prec b4+ y5+ b5+ y6+ b6+ b7+
KQWLQEVK+XL_L
B_b_ion
B_y_ion
intensity
m/z
0
200
400
600
200
400
600
800
1000
y2+ b2+ y3+ y6++ y4+ b3+ prec y5+ b4+ b5+ y6+ b6+ b7+
